# Global research trends of nanotechnology for pain management

**DOI:** 10.3389/fbioe.2023.1249667

**Published:** 2023-08-28

**Authors:** Yi Zhu, Yiyi Yao, Riyu Kuang, Zheng Chen, Zhen Du, Shuangquan Qu

**Affiliations:** Department of Anesthesiology, Hunan Children’s Hospital, Changsha, China

**Keywords:** nanotechnology, pain management, bibliometrics, visualisation, trends, VOSviewer, citespace

## Abstract

**Background:** Nanotechnology has been increasingly used in healthcare during recent years. However, the systematic evaluation of research on nanotechnology for pain management is lacking. In this study, we employed a bibliometric approach to examine the status of the research and global trends of nanotechnology in relation to pain management.

**Methods:** We selected relevant papers published in the Web of Science Core Collection database between 2013 and 2022 using search terms related to nanotechnology and pain management. Subsequently, the following bibliographic information was collected: publication year, originating country/region, affiliated authors and institutions, published journal, references cited, citation frequency, and keywords. The bibliometric software programs VOSViewer and CiteSpace were employed to obtain bibliometric statistics and perform visual analysis.

**Results:** A total of 2680 papers were retrieved. The number of publications in the field of nanotechnology for pain management has been increasing annually since 2013. China had the highest number of published papers, whereas the United States led in total citations. The Chinese Academy of Sciences was the most prolific institution, while the Tehran University of Medical Sciences had the highest overall citations. Furthermore, De Paula was the most prolific author. Papers associated with nanotechnology for pain management were mainly published in the *International Journal of Pharmaceutics*, *Pharmaceutics*, and the *International Journal of Nanomedicine*. Keyword analysis showed that “*in-vitro*” and “drug-delivery” appeared most frequently, with the top 10 common keywords comprising nanoparticles, pain, *in-vitro*, drug-delivery, delivery, release, inflammation, neuropathic pain, formulation, and expression. Lastly, the latest emerging keyword was “electrochemical sensor”.

**Conclusion:** Research on applying nanotechnology for pain management is growing steadily. China is the top country in terms of number of publications, with institutions under the Chinese Academy of Sciences making significant contributions to this field. “*In-vitro*” and “drug-delivery” are the current hotspots in this area, with “electrochemical sensor” as the latest topic at the research forefront. However, national and inter-institutional collaborations should be strengthened to enable patients with pain disorders to benefit from nanotechnology implementation in pain management.

## Introduction

Pain is an extremely common symptom observed in clinical practice. Patients with complex and challenging conditions that result in severe acute or chronic pain often experience serious physiological and psychological effects in various aspects of their life or work (Hylands-White et al., 2017; Cornish and Cornish, 2020; Fitzcharles et al., 2021). In terms of sensation, pain is an unpleasant emotional experience that can also cause mental distress. From a biological perspective, pain is an obvious signal of damage onset, indicating that a certain body part has been injured or is in imminent harm. Pain constitutes a physiological process in which certain molecules undergo signal transduction to produce pain responses (Wicksell et al., 2017; Wilson, 2017). Nociceptive, inflammatory, and neuropathic pain are the three general categories of pain. Although pain is a widespread and harmful phenomenon, its current treatment methods are relatively limited. Moreover, a patient with chronic pain will undergo overwhelming mental trauma, requiring pain alleviation via medication or other interventions (Hagiwara et al., 2020; Hoehn et al., 2022).

Common analgesics to treat chronic pain in clinical practice include non-steroidal anti-inflammatory drugs (NSAIDs) and morphine-based painkillers (Shaheed et al., 2020). However, many complications and side effects have resulted from the long-term and/or large-dose administration of these drugs. NSAIDs can cause stomach pain, nausea, or indigestion, while some patients may experience headaches, drowsiness, or insomnia. Furthermore, prolonged opioid use can lead to tolerance, dependence, and even respiratory depression in large doses (Vadivelu et al., 2018). The application of nanotechnology can effectively reduce the side effects of pain medications and mitigate tolerance development, thereby improving treatment efficiency. Different pain drugs can be loaded onto various nanocarriers, including natural, synthetic, and copolymer nanocarriers, for different medical interventions (Mendoza and Arruebo, 2020; Song et al., 2022). The development of nanotechnology has facilitated the emergence of pain relief strategies utilising various nanomaterials and targeted surface modifications. Furthermore, nanomaterials not only function as drug delivery carriers but can also be deployed to relieve chronic pain via the gradual release of pain medication, thus prolonging the duration of pain relief (Wu et al., 2020; Yu et al., 2022). Therefore, nanomaterials may provide many benefits for pain management. However, implementing nanotechnology in pain management requires further elucidation to yield the appropriate benefits to patients with pain conditions and even avoid potential adverse outcomes. Although scientists have investigated the use of nanotechnology in treating patients with pain disorders, the available literature on nanotechnology for pain management is not well integrated. Considering this issue, a systematic study is required to summarise the current state of research on nanotechnology applications in patients with pain disorders.

Bibliometrics is a popular and important scientific research tool applicable to all disciplines, including biomedicine (Chen and Song, 2019). Bibliometric analysis is a well-known research method based on mathematics and statistics used to evaluate academic productivity, reveal the status and hotspots in a particular discipline, and predict scientific trends in the study field according to the metrological characteristics of the relevant literature in various databases. This depth of analysis is not achievablein single-centre studies and reviews (Huang et al., 2022; Li L. et al., 2022; Li X. et al., 2022; Guan et al., 2023). Additionally, bibliometric software, such as VOSViewer, can be used to generate maps with high aesthetics and readability. Moreover, VOSViewer has a friendly user interface and is easy to operate and suitable for beginners. Another software tool CiteSpace offers a wealth of parameter settings and customisation options, allowing users to utilise flexible settings and perform analyses according to their own research needs. Furthermore, bibliometric and visual analyses enable the illustration of textual information in terms of the contributions and collaborations of different countries or regions, institutions, journals, and authors, improving the interpretability of this information (Liu L et al., 2022). Both these software were used in this study, enhancing the presentation and accuracy of the data analysis. As mentioned earlier, although research on nanotechnology for pain management is growing, no systematic analysis of the related literature is currently available. Therefore, this study provides a bibliometric and visual analysis of the literature on nanotechnology for pain management over the last decade (2013–2022), with an aim to improve the understanding of the present state of this research field, provide a forecast of topics of interest to scholars worldwide, and shed light on the future trends.

## Materials and methods

### Data sources

**FIGURE 1:**
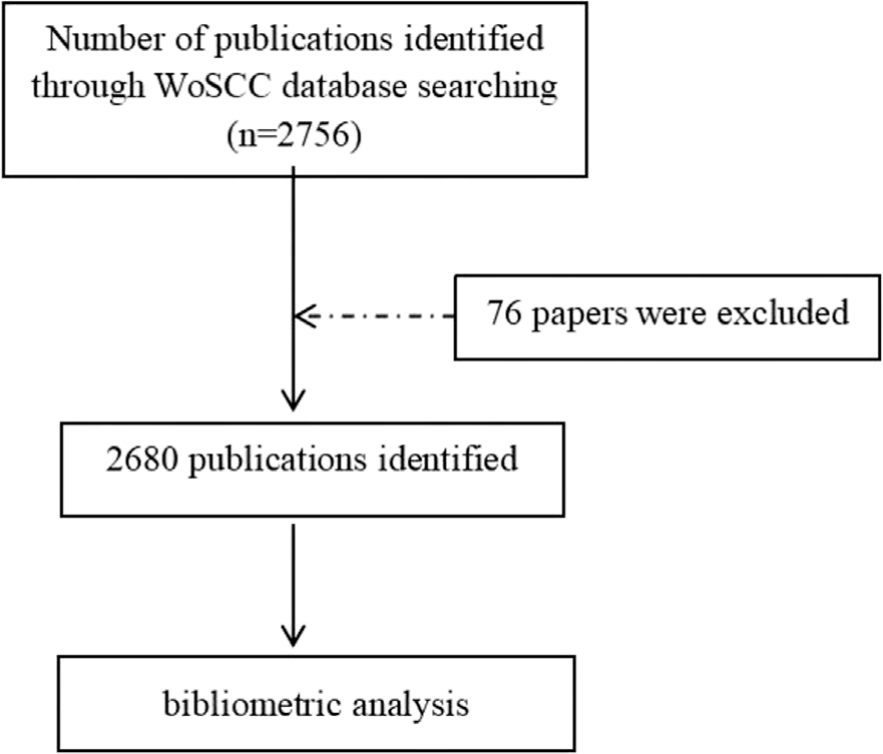
Flow diagram of literature identification.

The Web of Science (WoS) database, created by the Institute for Scientific Information, was used as the data source. The search words were set to TS = (Nano*) AND TS = (pain OR analgesia) AND PY = (2013–2022) to screen for literature on nanotechnology for pain management published in the past decade (2013–2022). The literature search was performed on 21 March 2023. All bibliographic data were stored in plain text and included information such as keywords, source country/region, author and institution affiliation, publication journal, references cited, and citation and keyword frequency (Figure 1). The restricted types of literature searched were treatises and reviews, excluding other literature types such as newsletters and abstracts.

### Data screening and visualisation

Two independent technical staff members conducted the literature screening and data collation separately. Annual publication trends, national/regional distribution, source journals, references, frequency of papers cited, and keywords were extracted and analysed from the screened literature. A bibliometric statistical and visual analysis was performed using VOSViewer 1.6.18 and CiteSpace 5.7.R2.

## Results

### Annual publication output

**FIGURE 2:**
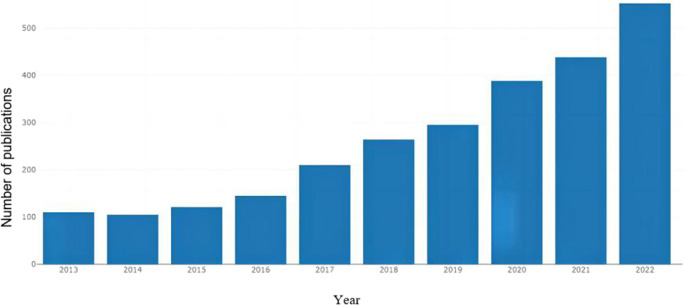
Annual trend of research publications on nanotechnology for pain management.

A total of 2680 eligible papers on nanotechnology for pain management published during the past decade (2013–2022) were retrieved from the WoS Core Collection (WoSCC) database (Figure 1), which received 53489 total citations. Among the 2680 publications, 1101 were articles, and 166 were reviews. As shown in Figure 2, the number of publications in the research field of nanotechnology for pain management has been increasing annually since 2013.

### National and regional productivity

**FIGURE 3:**
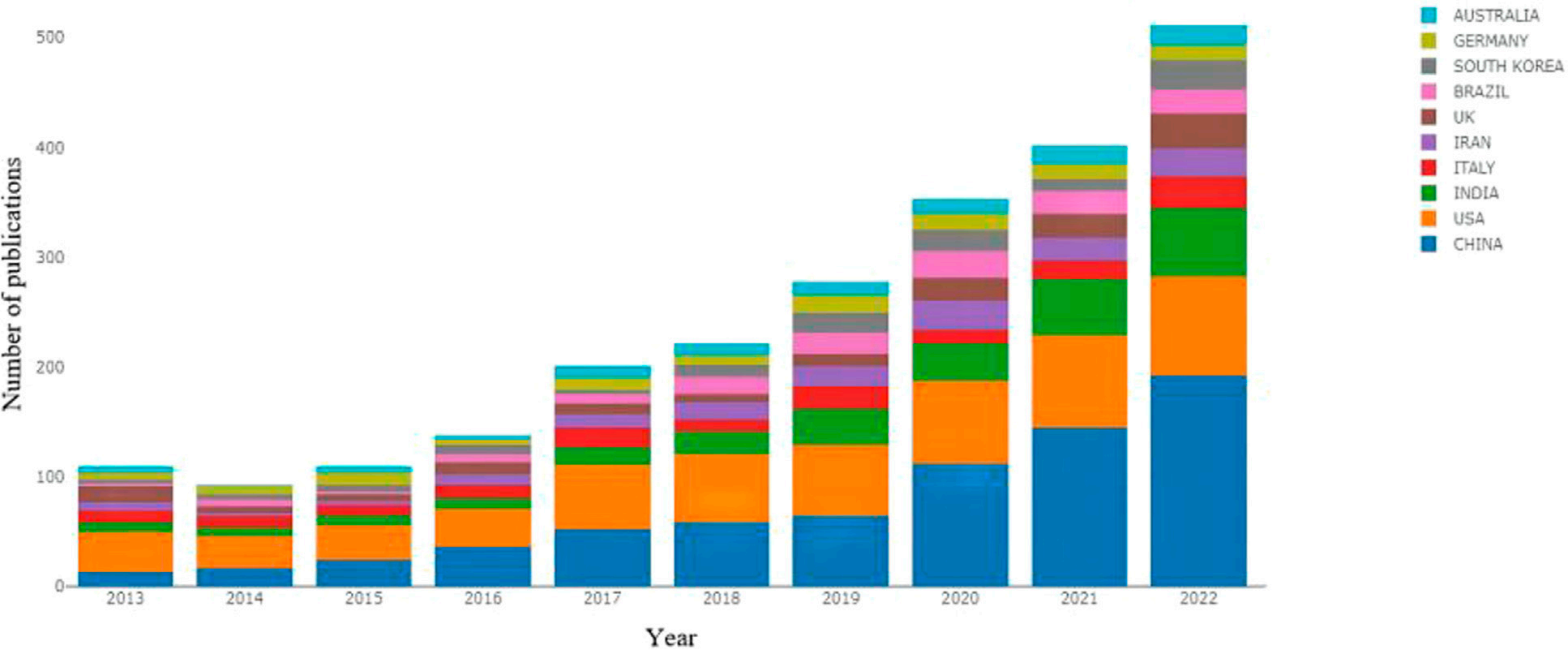
Annual publication trends among the top 10 countries in the field of nanotechnology applications for pain management.

**FIGURE 4:**
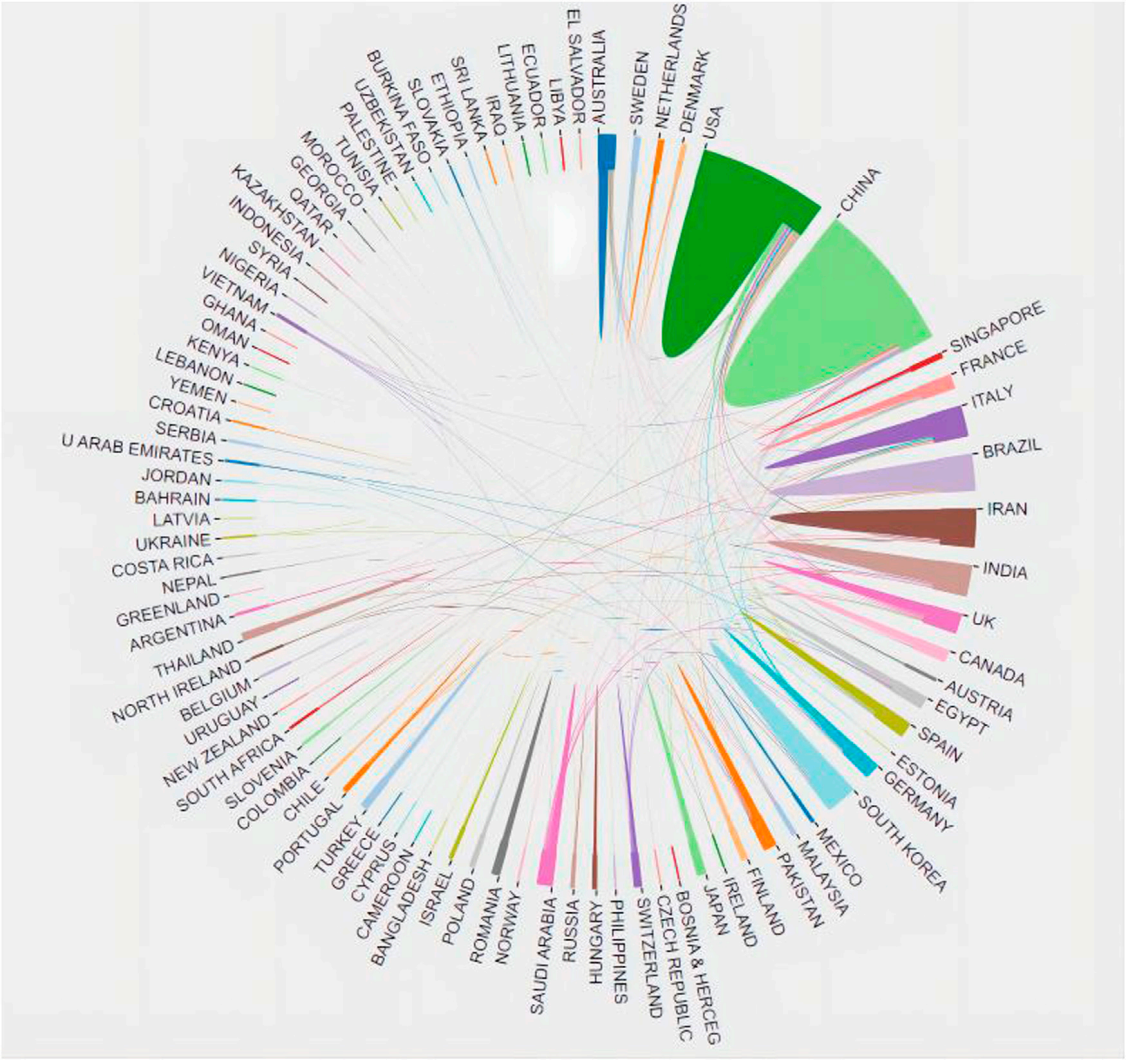
Publications and collaborations in the field of nanotechnology for pain management according to country/region.

A total of 95 countries/regions have published papers on nanotechnology applications in pain management. Table 1 lists the most productive countries/regions according to the number of publications. China was the most productive country (683 papers), followed by the United States (USA; 575 papers) and India (260 publications). The USA, China, and England were the most influential countries, with their papers being collectively cited 8729, 2214, and 1034 times, respectively. The annual trends in the publication output among the top 10 most productive countries are depicted in Figure 3. The distribution of papers by country and region is presented in Figure 4.

**TABLE 1:** Top 10 contributing countries in the field of nanotechnology for pain management.

Rank	Country	Publications	Citations	Total link strength
1	China	683	11154	223
2	United States of America	575	19955	368
3	India	260	4219	111
4	Italy	152	3463	148
5	Iran	145	2766	78
6	Brazil	133	8786	62
7	England	124	10334	146
8	South Korea	116	2445	74
9	Germany	107	9953	123
10	Australia	101	2469	112

### Institutional contributions

**FIGURE 5:**
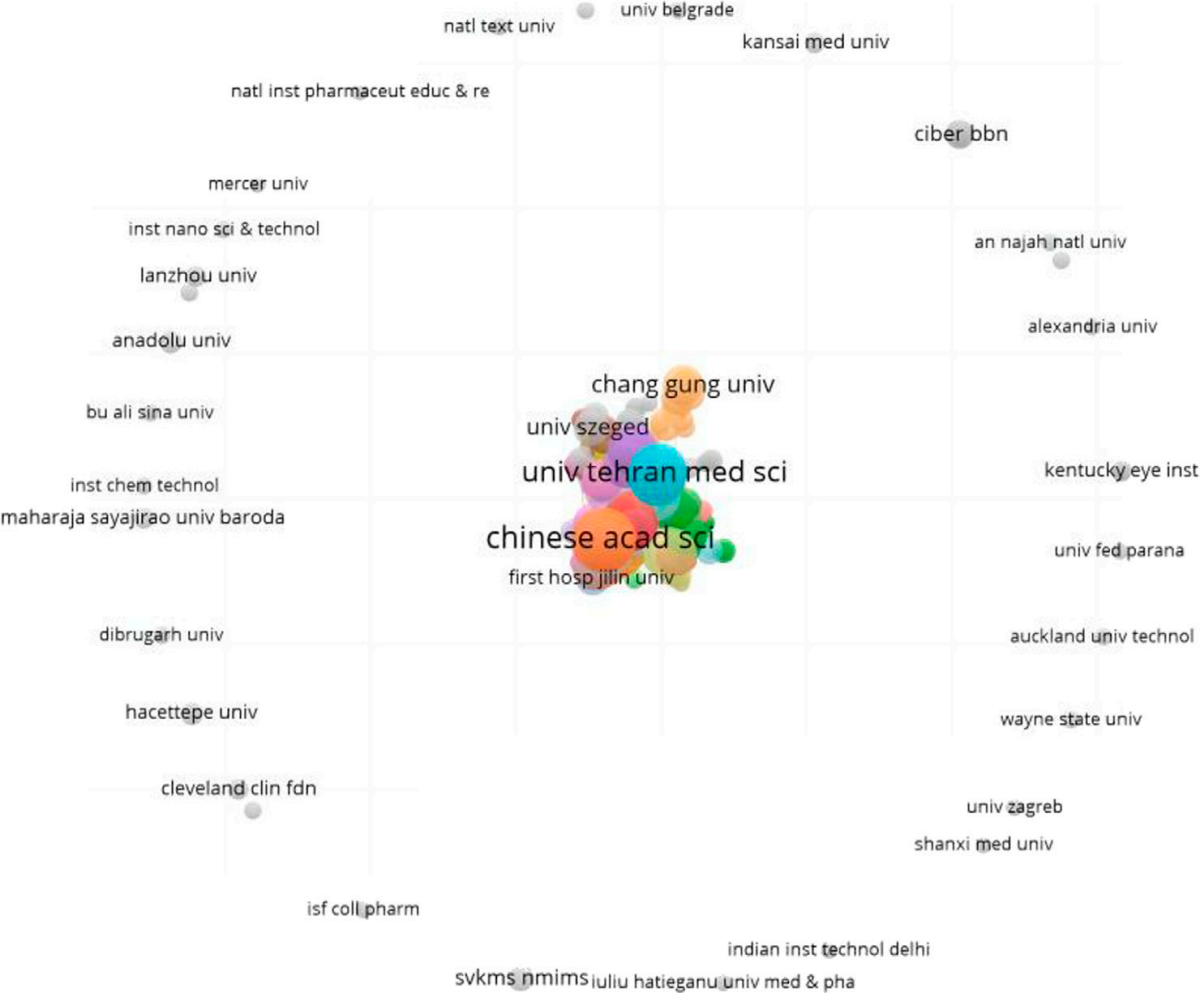
Institutional contributions in the research field of nanotechnology for pain management.

A total of 3492 different institutions were involved in the research on nanotechnology for pain management, with at least five papers published by 273 institutions. The top 10 most active institutions are listed in Table 2, including six in China and two in Iran. The Chinese Academy of Sciences (China) was found to be the most productive institution, with 39 published papers. The next two institutions were (Sichuan University in China and the Tehran University of Medical Sciences in Iran), contributing 31 publications each. The Tehran University of Medical Sciences, the Chinese Academy of Sciences, and Jilin University were the most influential institutions, with their papers receiving 845, 793, and 716 citations, respectively. Figure 5 presents the distribution and collaboration of the institutions.

**TABLE 2:** Top 10 institutions with the most publications.

Rank	Organisation	Publications	Citations	Total link strength
1	Chinese Academy of Sciences	39	793	58
2	Sichuan University	31	477	14
3	Tehran University of Medical Sciences	31	845	22
4	Islamic Azad University	30	465	17
5	Shanghai Jiao Tong University	30	524	41
6	The University of Queensland	30	465	19
7	Seoul National University	27	540	24
8	Zhejiang University	26	293	16
9	Jilin University	24	716	31
10	University of Chinese Academy Sciences	24	426	54

### Authors’ contributions

**FIGURE 6:**
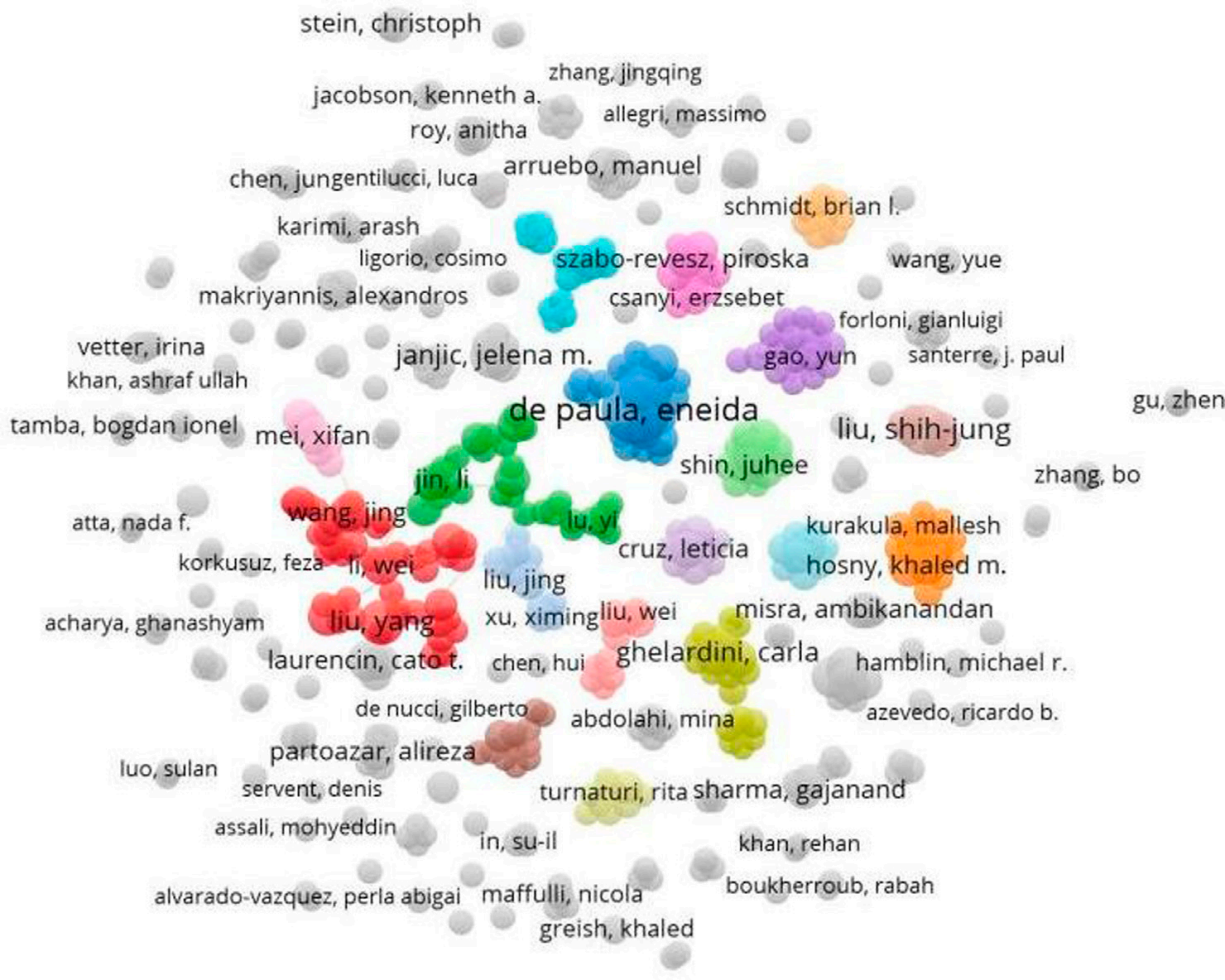
Collaboration network map of authors in the research field of nanotechnology for pain management.

A total of 15186 authors contributed to the research on nanotechnology for pain management. The visual analysis of the core authors is shown in Figure 6, while the top 10 authors with the highest number of publications are listed in Table 3. The analysis revealed that De Paula, Shih-Jung, and Yang were the most prolific authors, accounting for 22, 12, and 10 publications, respectively. In terms of citation frequency, De Paula, Ghelardini, and Laurencin were the most cited authors, with their papers receiving 373, 216, and 163 citations, respectively.

**TABLE 3:** Top 10 authors with the highest number of publications.

Rank	Author	Publications	Citations	Total link strength
1	De Paula, Eneida	22	373	74
2	Liu, Shih-Jung	12	126	20
3	Liu, Yang	10	137	7
4	Janjic, Jelena M	10	122	14
5	Kim, Dong W	10	102	45
6	Ghelardini, Carla	9	216	27
7	Ribeiro, Ligia NM	9	139	43
8	Laurencin, Cato T	8	163	12
9	Du, Wei	8	86	26
10	Hosny, Khaled M	8	83	42

### Journal analysis

**FIGURE 7:**
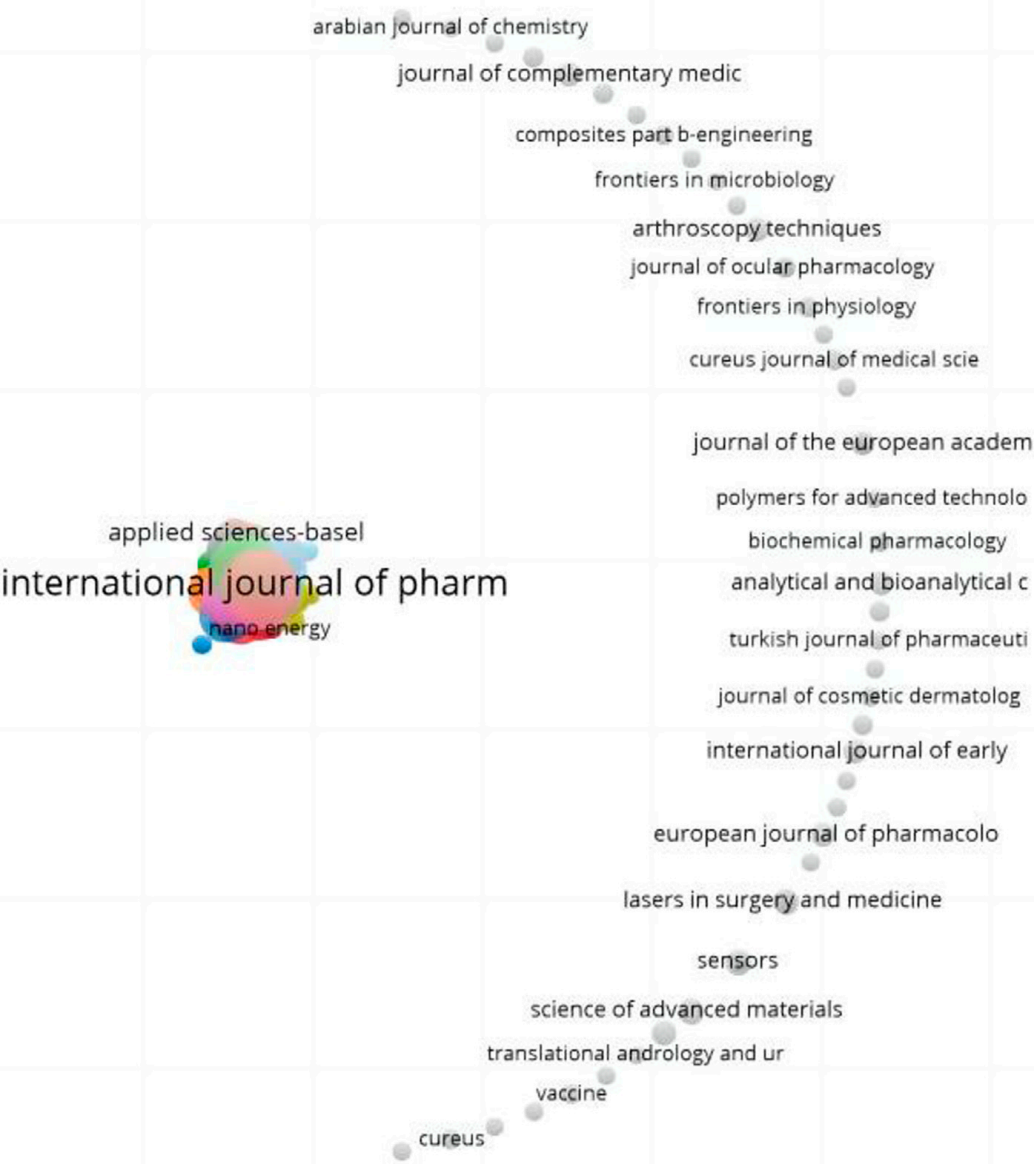
Collaborative network of journals in the research field of nanotechnology for pain management.

A total of 982 journals have published papers related to nanotechnology for pain management, and the visual analysis of major journals is shown in Figure 7. The *International Journal of Pharmaceutics* was the most active, with 29 published papers. The next two journals were *Pharmaceutics* and the *International Journal of Nanomedicine*, publishing 17 and 16 papers, respectively (Table 4).

**TABLE 4:** Top 10 journals with the most publications in research on nanotechnology for pain management.

Rank	Journal	Publications	Citations	IF2022	JCR
1	*International Journal of Pharmaceutics*	49	986	5.8	Q1
2	*Pharmaceutics*	43	646	5.4	Q1
3	*International Journal of Nanomedicine*	38	731	8	Q1
4	*International Journal of Molecular Sciences*	36	419	5.6	Q1
5	*Journal of Drug Delivery Science and Technology*	36	343	5	Q1
6	*Acta Biomaterialia*	34	1295	9.7	Q1
7	*Scientific Reports*	33	659	4.6	Q2
8	*Molecules*	27	494	4.6	Q2
9	*Biomaterials*	24	996	14	Q1
10	*Drug Delivery*	24	394	6	Q1

### Keyword analysis

**FIGURE 8:**
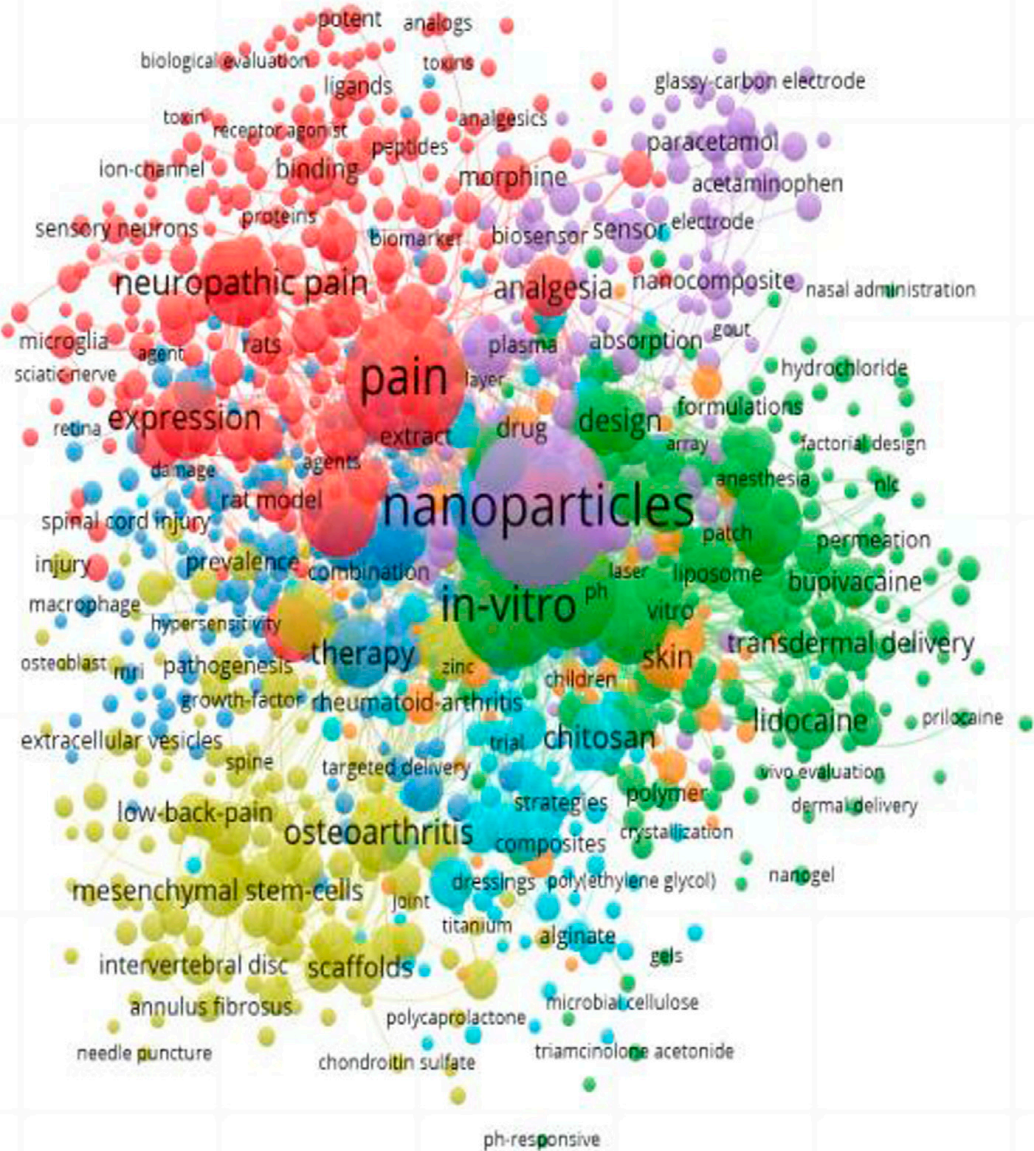
A co-occurrence network diagram of the keywords.

**FIGURE 9:**
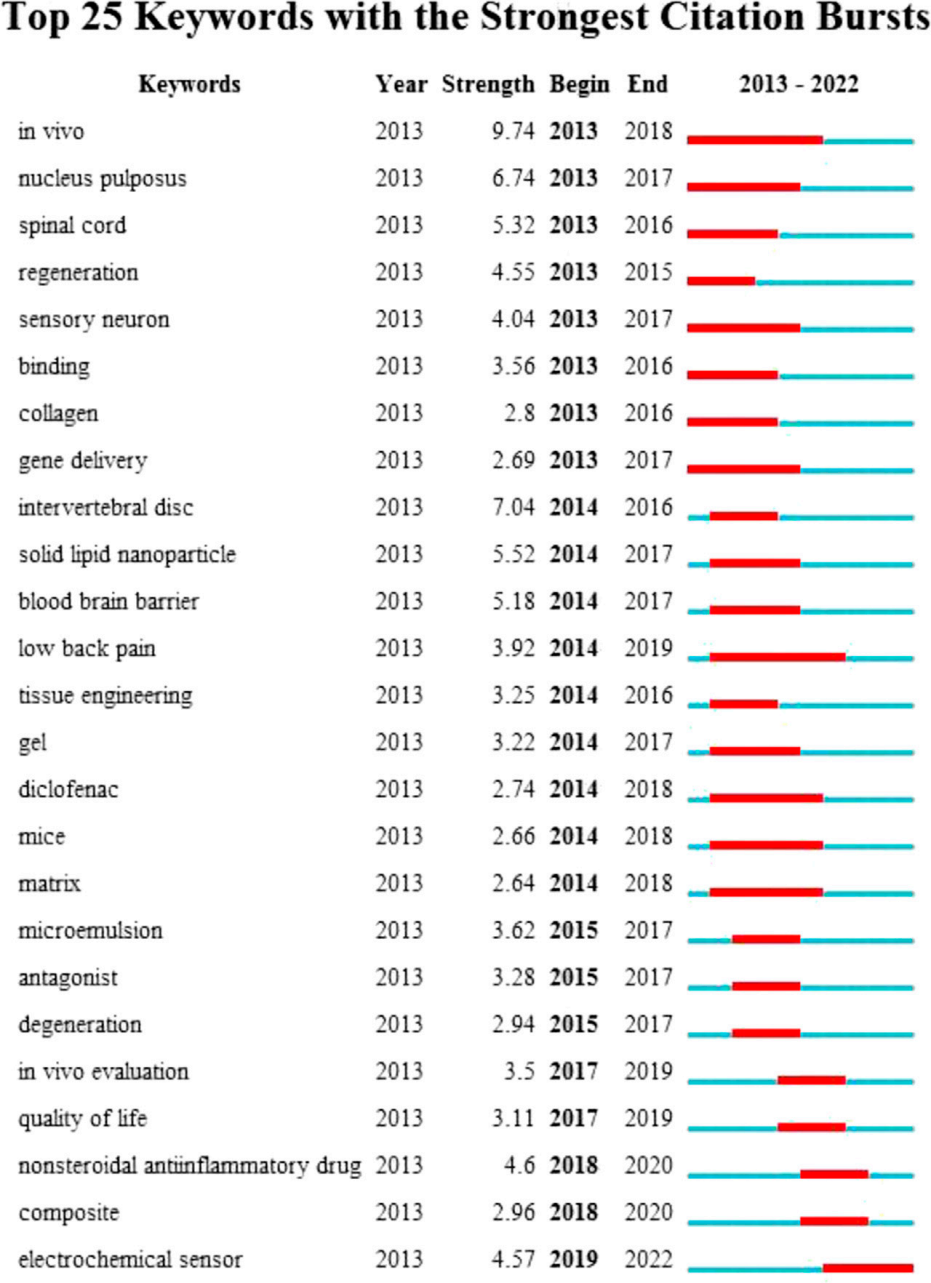
Emergent analysis of the keywords.

Keywords are condensed representations of the literature in a particular field, with high-frequency keywords in multiple papers considered research hotspots. The keywords in the identified literature were summarised and filtered for high-frequency sub-terms. The resulting keywords are illustrated in Figure 8. The statistical analysis revealed 13033 keywords in 2680 papers. The top 10 most frequently occurring keywords, including nanoparticles, pain, *in-vitro*, drug-delivery, delivery, release, inflammation, neuropathic pain, formulation, and expression, are displayed in Table 5. After the subject terms, ‘*in-vitro*’ was the most frequently used keyword (320 co-occurrences), followed by ‘drug-delivery’ (250 co-occurrences), consistent with the theme of our study. Figure 9 shows the results of a keyword emergent analysis. The most recent keyword to standout was ‘electrochemical sensor'. Further analysis shows that early research focused on the analgesic effect of nanoparticles at the spinal cord level, and then the research hotspot shifted to the pain-relieving effect of nano-formulations of non-steroidal anti-inflammatory drugs (NSAIDs), and in the last few years scholars have paid more attention to the hotspot of electrochemical sensing technology.

**TABLE 5:** Top 10 commonly used keywords in publications on nanotechnology for pain management.

Rank	Keyword	Frequency	Total link strength
1	Nanoparticles	522	3761
2	Pain	393	2437
3	*In-vitro*	320	2346
4	Drug-delivery	250	1893
5	Delivery	209	1557
6	Release	168	1301
7	Inflammation	161	1164
8	Neuropathic Pain	149	1018
9	Formulation	136	1137
10	Expression	119	797

## Discussion

The rapid development of nanotechnology has been accompanied by the establishment of medicine as an important area of nanotechnology applications. However, nanotechnology implementation in pain management is only in its nascent stage. The biggest challenges in pain management using medications comprise their limited duration of action and side effects (Chakravarthy et al., 2017; Bidve et al., 2019). The adoption of nanotechnology has helped patients with pain disorders to access comparatively better healthcare because nanotechnology systems overcome many problems encountered in using conventional analgesics. The use of nanomaterials and nanoparticles can achieve pain management at the molecular level, leading to improved outcomes, reduced analgesic drug doses, and enhanced long-term efficacy and safety (Chen et al., 2020; Da Silva et al., 2022; Dash and Kundu, 2023). In recent years, nanotechnology research for pain management has increased, playing a crucial role in advancing the relevant nanotechnology strategies (Kopach et al., 2018; Kuthati et al., 2020). From 2013 to 2022, 2680 papers have been published. However, a systematic analysis and summary of the research and literature in this domain have been lacking. Furthermore, the development of computer search techniques and the growth of bibliometric analysis enable us to digitally retrieve and analyse information concerning the research findings on nanotechnology for pain management. In this study, current literature data on nanotechnology for pain management was analysed bibliometrically and visually from an information science perspective.

China is the leading country with the highest number of published papers, indicating that it is at the forefront of research on nanotechnology for pain management. This observation may be attributed to the health strategy implemented by China. Moreover, China has a government-led and policy-supportive approach in the field of nanotechnology, promoting the development of nanotechnology through financial investments and talent recruitment. With the support of government funding, the research, development, and industrialisation of nanopain medicine technology have gradually developed, leading to the growth of many prominent companies and research teams (Duschl and Windgasse, 2018). In the case of the USA, nanotechnology research was initiated comparatively earlier; therefore, this field has a strong basic research foundationin this country. Currently, the USA focuses more on technical research and commercialisation in this domain, supporting the development of the nanotechnology industry via research funding and national laboratories. Additionally, the USA has some of the world’s leading research institutions, with excellent research conditions as well as laboratory equipment and tools to foster high-quality nanomedicine research. Moreover, the USA has a strong industrial base in the medical devices, pharmaceuticals, and biotechnology sectors, and these industries strongly support nanomedicine research (Shen et al., 2017; Qiuyue et al., 2023). Lastly, nanotechnology use in the USA patient population with pain conditions is relatively well established. Although the field of nanotechnology for pain management has attracted worldwide attention, co-operation between different countries in this research field is limited. Therefore, international co-operation within this domain requires to be strengthened.

The number of publications, citations, and keyword co-occurrences is an important indicator of the centrality and scientific collaborations of an institution in a particular research field as well as reflects the academic level of the country where the institution is located. Here, six of the top 10 institutions in research on nanotechnology for pain management were from China, confirming China’s prominence in this field. The Chinese Academy of Sciences in China was the most prolific institution, with 28 publications and the highest number of total citations. Of the 15,186 authors contributing to the research on nanotechnology for pain management, De Paula was the most influential author, with 22 published manuscripts and 373 citations overall. In the case of the journals in this domain, most publication journals were medical journals. Papers on nanotechnology for pain management were mainly published in the *International Journal of Pharmaceutics, Pharmaceutics,* and the *International Journal of Nanomedicine*. These highly productive journals have contributed to the global development of the field of nanotechnology for pain management.

Keyword analysis is a vital aspect of bibliometric analysis, summarising the paper content and visually expressing the research direction and publication topic. Nanotechnology can benefit patients with pain conditions, and the most frequent keywords in our analysis were ‘*in-vitro*’ and “drug-delivery”, suggesting that nanotechnology-based drug-delivery in pain management is a significant research topic. The employment of nanotechnology for drug delivery can help address the issues of limited duration of action and side effects of the existing pain medications. Nanocarriers have been demonstrated to facilitate drug delivery to the target site at an appropriate level, thereby reducing systemic toxicity (Berrocoso et al., 2017; Hua and Wu, 2013). Specific drugs delivered in nanocarriers for pain relief include local anesthetics, nonsteroidal anti-inflammatory drugs, opioids, and others. Thus, nanotechnology use for drug delivery may improve therapeutic efficiency, reduce drug side effects, and mitigate tolerance development. Different pain medications have been loaded onto different nanocarriers, including natural, synthetic, and copolymer nanocarriers, for various medical purposes (Khazraei et al., 2017; Wang et al., 2022). Pain relief strategies using varied nanomaterials and targeted surface modifications have grown rapidly with nanotechnology advances. Burst terms are new words or terminologies that exhibit a rapid increase in frequency in a specific topic or field within a certain period, effectively reflecting the hot changes in that particular research area. In this study, “electrochemical sensor” was identified as a recent burst term, indicating that this topic is an emerging research Frontier in the field of nanomaterials for pain management and warrants further investigation. Furthermore, nanoparticles can be used to detect the source of pain signals. The injection of nanoparticles locally into the pain site may help to accurately identify the source of pain molecules by localising and tracking neurotransmitter secretion *in vivo* (Tronino et al., 2016; Khanna et al., 2021; Qiao et al., 2023). This approach enables the precise targeting of pain treatment, representing the latest advancements in nanotechnology in this field. Due to various constraints, most of the existing studies on nanocarriers in the field of pain management are still at the preclinical research stage, and the more researched ones that are now on the market include Exparel, a bupivacaine liposome-infused suspension, which has been approved by the FDA in the United States.

Although nanotechnology applications in pain management have developed greatly, several challenges and limitations remain. A few issues that require further investigation include ensuring the reliability and safety of nanotechnology for pain management and integrating nanotechnology with traditional medical practice. Additionally, nanotechnology use in pain management requires close collaboration between specialists and other support staff. Attention also needs to be paid to the transparency and interpretability of nanotechnology to enable physicians and patients to comprehend and accept the results obtained from implementing nanotechnology. Nevertheless, applying nanotechnology in pain management appears promising and may improve outcomes and quality of life for patients with pain disorders.

Although nanotechnology offers many advantages, combining pain medication with nanotechnology is relatively expensive, resulting in certain difficulties in its widespread adoption across different countries and regions worldwide. Nevertheless, future developments in nanotechnology will facilitate its widespread deployment. Moreover, nanotechnology for pain management should be considered an evolving process, and the choice of nanotechnology should factor in the analgesic effects and economic costs. Overall, nanotechnology use in pain management has wide application potential and far-reaching implications. Furthermore, the continuous development and improvement in nanotechnology will serve as an important driving force in pain management research, enhancing the health security and quality of life in patients with pain conditions worldwide.

## Limitations

This study has several limitations that should be considered. First, the bibliometric analysis results are influenced by the data source, with certain variations in the literature included in each database. The database we used was WoSCC; therefore, the publications in non-English language databases were not included. Nonetheless, the WoSCC database is a leading global literature database with high-quality literature across the world, making it an ideal database for bibliometric studies. Second, bibliometric research relies heavily on the citation relationships of published literature to infer collaborations among scholars and connections between research fields. However, the citation behaviour of researchers is influenced by numerous factors, including author status, publisher reputation, and journal impact factor. Thus, bias may occur when analysing social networks and disciplinary intersections in bibliometric examination.

## Conclusion

Our study is the first to visually analyse the current state and future trends in nanotechnology research for pain management. Overall, this research field is in a rapidly evolving phase. China is the leading research country, with institutions affiliated with the Chinese Academy of Sciences having significant contributions. ‘*In-vitro*’ and “drug-delivery” are the current research focus, and ‘electrochemical sensor’ is at the research forefront. However, national and inter-institutional collaboration should be strengthened to enable patients with pain disorders to benefit from nanotechnology advancements. This study will serve as a useful reference for further research on nanotechnology applications for pain management.

## Data Availability

The original contributions presented in the study are included in the article, further inquiries can be directed to the corresponding author.
